# HER2-PI9 and HER2-I12: two novel and functionally active splice variants of the oncogene HER2 in breast cancer

**DOI:** 10.1007/s00432-021-03689-1

**Published:** 2021-06-16

**Authors:** Vic Hart, Marco Silipo, Swapna Satam, Hannah Gautrey, John Kirby, Alison Tyson-Capper

**Affiliations:** Translational and Clinical Research Institute, Faculty of Medical Sciences, Newcastle University, Newcastle upon Tyne, NE2 4HH UK

**Keywords:** HER2, Splice variants, Breast cancer, Trastuzumab, Lapatinib, HER2-∆16, P100, Herstatin

## Abstract

In this study, two novel alternative splice variants of HER2, named HER2-PI9 and HER2-I12, were identified in breast cancer cell lines and breast tumour tissues. Whilst HER2-P19 arises from the inclusion of an 117 bp cassette-exon of intron 9 of HER2, HER2-I12 results from intron 12 inclusion. In silico analyses were performed to predict the amino acid sequences of these two HER2 novel variants. To confirm their protein expression, plasmid vectors were generated and transfected into the HER2 negative breast cancer cell line, MCF-7. Additionally, their functional properties in oncogenic signalling were confirmed. Expression of HER2-PI9 and HER2-I12 was successful and matched the in silico predictions. Importantly, these splice variants can modulate the phosphorylation levels of extracellular signal-related kinase 1/2 (ERK1/2) and Akt/protein kinase B (Akt) signalling in MCF-7 breast cancer cells. Enhanced cellular proliferation, migration and invasion were observed in the case of the HER2-I12 expressing model. In human tissues and breast carcinoma tumours both variants were present. This study reveals two novel splice variants of HER2. Additionally, the potential biological activity for HER2-PI9 and HER2-I12 in breast cancer cells is also reported..

## Background

The human epidermal growth factor receptor 2 (HER2) is an oncogenic member of the epidermal growth factor receptor family. Overexpression of HER2 or amplification of the encoding gene, *ERBB2,* defines a subset of breast cancer typically representing 15–20% of all cases (Oh and Bang 2020; Ferrari et al. 2016). This receptor transduces biochemical signals across the cell membrane alongside EGFR, HER3 and HER4 (Dittrich et al. 2014). This family is activated by a range of ligands including growth factors and heregulins (Singh et al. 2016). Activation triggers homo- or hetero-dimerization and a shifting of the receptor domains to allow for auto-phosphorylation of C-terminal tyrosine residues (Bragin et al. 2016). These become docking sites where downstream signalling molecules with Src homology 2 domains are subsequently activated (Ferguson 2008; Jadwin et al. 2018). HER2 is the favoured dimerization partner due to its signal amplification abilities, despite being an ‘orphan receptor’ (Aertgeerts et al. 2011). All members of the family may dimerize despite differing structures (Arkhipov et al. 2013; Peckys et al. 2019).

The two key signalling pathways associated with HER2 oncogenic properties are the rat sarcoma/mitogen-activated protein kinase (RAS/MAPK) pathway and the phosphatidylinositol 3-kinase/protein kinase B (PI3K/Akt) pathway (Aksamitiene et al.^2012^; Yang et al. 2016; Ruiz-Saenz et al. 2018). Key cellular functions affected include proliferation, apoptosis, cell cycling, metabolism, protein synthesis, angiogenesis, stemness and epithelial-mesenchymal transitions (Barros et al. 2010; Nikolai et al. 2016; Madonna et al. 2019; Liu et al. 2014; Geng et al. 2014). HER2 maintains a constitutively active shape and therefore can continually stimulate signalling pathways (Dittrich et al. 2014). Metastatic spread to the bone, brain, liver and lungs is common (Wang et al. 2017; Kennecke et al. 2010). Thus, HER2 overexpression has major repercussions for the development of aggressive cancer phenotypes.

HER2 has proven useful as both a prognostic marker and therapeutic target in breast cancer. Immunohistochemistry (IHC) and in situ hybridization clinical tests are used to predict a score for each patient, based on the extent of receptor expression and gene amplification (Tsai et al. 2019). If a patient is classified as HER2 positive (HER2 +) then HER2 targeted therapies are prescribed and can be administered in tandem with chemotherapeutics, surgery and endocrine treatment (Cardoso et al. 2018). Standard-of-care treatments include combinations of the monoclonal antibodies trastuzumab and pertuzumab which target distinct extracellular regions of the HER2 protein (Choong et al. 2020). These block receptor-activated cell signalling and induce an immune response (Nami et al. 2019). Lapatinib is a small molecule inhibitor which targets the ATP-binding site of HER2 and EGFR (Bauerfeind et al. 2010). T-DM1 is a combined treatment of Trastuzumab and the cytotoxic molecule DM1 and is used as a second-line treatment (Cesca et al. 2020; Marti et al. 2020).

Pre-mRNA splicing allows for the removal of introns from nascent RNA and the joining of exons to produce a mature RNA transcript (Nilsen and Graveley 2010). Owing to the widespread impact of splicing, oncogenes frequently express alternative splice variants (Bonnal et al. 2020). Aberrant splicing patterns produce functional and non-functional end-products which have been identified in many cancers, including breast (Sveen et al. 2016; Zhao et al. 2016). These is turn have important effects on the hallmarks of cancers (Oltean and Bates 2014).

Different forms of HER2 proteins are present in breast tumours, some being produced via alternative splicing (Jackson et al. 2013; Hart et al. 2020). HER2 wild-type (HER2-WT) consists of an extracellular region, a transmembrane domain that crosses the cell membrane, and an intracellular region with a C-terminus (Peckys et al. 2019). HER2-∆16 is produced via an in-frame deletion of a small cassette exon which generates a HER2 protein lacking 16 amino acids in the juxtamembrane region (Castagnoli et al. 2019). This produces strong covalent bonds between dimers resulting in consistent activation and presence at the cell surface (Turpin et al. 2016; Alajati et al. 2013). HER2-∆16 enhances Src signalling, mediating its heightened oncogenic properties (Mitra et al. 2009; Castagnoli et al. 2014). These include enhancing proliferation, migratory and invasive capabilities, and mediating stem-like properties and epithelial to mesenchymal transition (Palladini et al. 2017; Castagnoli et al. 2017). The modified structure and contribution to HER2 signalling addiction is linked with altered sensitivity to anti-HER2 treatments like trastuzumab and lapatinib (Castagnoli et al. 2014; Tilio et al. 2016).

Herstatin is a secreted ‘auto-inhibitor’ with intron 8 retention which causes truncation, producing a HER2 protein with only the extracellular regions and a novel C-terminus (Silipo et al. 2017). Herstatin can still bind to a HER2-WT receptor which stops HER2-WT homo- or hetero-dimerization and so blocks downstream signalling (Azios et al. 2001; Koletsa et al. 2008; Hu et al. 2005). This reduces cell survival, proliferation and impacts upon other oncogenic properties (Justman and Clinton 2002).

P100 is another truncated HER2 variant produced by the retention of intron 15 which includes a premature termination codon and poly (A) addition site which triggers truncation (Jackson et al. 2013; Gautrey et al. 2015). Expression reduces ERK1/2, a key node in the RAS/MAPK pathway, phosphorylation and cellular proliferation in vitro (Aigner et al. 2001).

There is evidence for the existence of further splice variants in breast cancer (Zhao et al. 2016; Dvinge and Bradley 2015). In this study, two novel HER2 variants are identified and assessed. HER2-PI9 and HER2-I12 arise from the inclusion of a cassette exon in intron 9 and inclusion of intron 12, respectively. Moreover, these are shown to be translated into proteins that are present in tumour and normal breast tissues and can activate downstream oncogenic signalling.

## Methods

### Breast cancer cell lines and tissues

All breast cancer cell lines were obtained from ATTC and are guaranteed authentic (ATCC, LGC Standards, Middlesex, UK) and cultured at 37 °C with 5% CO_2._ The line SKBR3 (HER2 + /oestrogen receptor–(ER)/progesterone receptor–(PR)) was cultured using McCoy’s 5a GlutaMAX medium (GIBCO by Life Technologies, Paisley, UK) supplemented with 10% Fectal Bovine Serum (FBS; Sigma-Aldrich, Dorset, UK) and 1 μg/ml Penicillin/Streptomycin (Sigma-Aldrich, UK). The line MCF-7 (HER2 −/ER + /PR +) was cultured using phenol red-free Dulbecco’s Modified Eagle medium (DMEM) (Sigma-Aldrich, UK) supplemented with 10% FBS (Sigma-Aldrich, UK), 2 mM l-Glutamine (Sigma-Aldrich, UK), and 1 μg/ml Penicillin/Streptomycin (Sigma-Aldrich, UK). MDA-MB-231 (HER2 −/ER −/PR −) was cultured in Leibovitz’s L-15 Medium (GIBCO by Life Technologies, UK), supplemented with 10% FBS (Sigma-Aldrich, UK), and 1 μg/ml Penicillin/Streptomycin (Sigma-Aldrich, UK). T47D (HER2 −/ER + /PR +) was cultured in RPMI-1640, phenol red free (Sigma-Aldrich, UK), supplemented with 10% FBS (Sigma-Aldrich, UK), and 1 μg/ml Penicillin/Streptomycin (Sigma-Aldrich, UK).

Grade 3 invasive ductal carcinoma (IDC) primary breast tumours were used in this study, these were previously classified as HER + /ER −; HER2 −/ER + ; HER −/ER + or HER-/ER-. Normal breast tissue samples from breast reduction surgery were also included; all tissues were obtained through the Breast Cancer Now Tissue Bank (BCNTB000027). Muscle, heart, liver, testis and cerebellum cDNA was purchased from Clontech Laboratories, Inc. (now Takara Bio USA, Inc, California, USA).

### Cellular transfections

Expression vectors were produced using a HER2 expression vector, a gift from Mien-Chie Hung (Addgene plasmid #16,257; http://n2t.net/addgene:16257; RRID:Addgene_16257, Massachusetts, USA). The HER2 sequence was cloned into pcDNA3.1 ( +) N-terminal FLAG-tag vector (96 Proteins, San Francisco, USA). The insert of the cassette-exon or intron 12 produced N-FLAG HER2-PI9 and N-FLAG HER2-I12. The vectors contain resistance to the antibiotic Geneticin (G418, Promega, Southhamton, UK).

MCF-7 cells were cultured to 80% confluency, washed with Dulbecco’s Phosphate-Buffered Saline (PBS) (Sigma-Aldrich, UK) and adherence disrupted with Trypsin–EDTA Solution (0.25%) (Sigma-Aldrich, UK). Cells were seeded at 1.2 × 10^5^ cells/well in a 12 well plate (Greiner Bio-one, Gloucestershire, UK) and transfected 24 h after seeding. 24 h later, 2 µg DNA, 4 µl Fugene®HD transfection reagent (Promega, UK) and 40 µl Opti-MEM® 1x + GlutaMAX™ (GIBCO by Life Technologies, UK) were added to the cells and incubated at 37 ℃ and 5% CO_2_ for 48 h before protein isolation. Western immunoblotting was performed, as described below, to confirm protein expression after each transfection.

To establish stably expressing cell lines the transient transfection protocol detailed above was followed. A kill curve preformed on the MCF-7 cell line identified 900 µg/ml as optimal G418 dose (Promega, UK). Transfections were performed as above in 80% confluent T25 flasks (Greiner Bio-one, UK). Non-transfected MCF-7 s were included for confirmation of unsuccessfully transfected cell die-off. At 48 h, selection media was applied; phenol red-free DMEM (Sigma-Aldrich, UK) supplemented with 10% FBS (Sigma-Aldrich, UK), 2 mM l-Glutamine (Sigma-Aldrich, UK) and 900 µg/ml G418 (Promega, UK). After 72 h cells were washed with PBS and selection media re-applied. At day 10 no cells remained in the 6-well plate indicating selection was complete. Transfected cells were passaged with Trypsin–EDTA Solution (0.25%) (Sigma-Aldrich, UK) and seeded at 0.5cells/well in 96 well plates. Wells containing a single cell were marked and expanded to produce monoclonal cell lines.

### Functional cellular assays

Stably transfected MCF-7 cells were tested in triplicate for all functional assays with three technical repeats per experiment. To assess proliferation, both a XTT assay and trypan blue assay were completed. For the XTT assay, 8000 cells/well were seeded in a 96 well plate (Greiner Bio-one, UK). 24 h after seeding 50 µl cell proliferation kit II (XTT, Roche Diagnostics, Mannheim, Germany), was added to each well. After a further 24 h, the plate was read at an absorbance of 450 nm and 650 nm on the Synergy HT plate reader (BioTech, Aligent, Didkot, UK) to calculate colour fold change.

For the trypan blue assay, cells were seeded in 12 well plates (Greiner Bio-one, UK), at 1.2 × 10^5^ and left overnight to adhere. Cells were counted at 72 h. Trypan blue solution (Sigma-Aldrich, UK) was mixed with cell solution 1:1. Cells were counted then viability calculated with the LUNA II automated cell counter (Logos Biosystems, Gyeonggi-do 14,055, South Korea).

A ‘wound-healing’ scratch assay was undertaken by seeding a 24 well plate (Greiner Bio-one, UK), at 2.4 × 10^5^ cells/well. At 48 h a 200 µl pipette tip as used to produce a scratch. Photos were taken at 0 and 48 h to assess % closure.

To assess migration, stable cell lines were seeded in the upper chamber of Corning^®^ Transwell^®^ polycarbonate membrane cell culture inserts, 6.5 mm Transwell with 8.0 μm pore (Corning, Kennebunk 04043, Maine, USA). Cells were serum starved for 24 h. Media with 20% FBS (Sigma-Aldrich, UK) was then added to bottom chamber as a chemoattractant. After 24 h, membranes were removed and cells fixed in 70% methanol at 4 °C, overnight. Cells that had migrated completely were counted with a trypan blue assay, as described above. The fixed Transwell inserts were stained for two minutes with Mayer’s Hematoxylin Solution (Sigma-Aldrich, UK) and dehydrated through a series of ethanol concentrations, 70%, 95% and 99%. Five images were taken of each stained insert and the cells counted on image J (Schneider et al. 2012) and an average taken.

To assess invasion, a migration assay protocol was followed with a coating of Corning^®^ Matrigel^®^ Basement membrane matrix, phenol red-free, LDEV-free (Matrigel^®^) (Corning, USA) and serum free media at a ratio of 1:4. 30 μl was added atop the transwell insert and the plate stored for 30 min at 37 °C before cell seeding.

Stable cell line models were placed in 3D culture. 100 μl Matrigel^®^ (Corning, USA) was used to coat a 24 well plate (Greiner Bio-one, UK). A final cell density of 3 × 10^5^ was embedded in 10% Matrigel^®^ (Corning, USA) solution in complete media. A complete media layer was placed over the embedded cultures and replaced every two days. Pictures were taken at 1, 3, 7 and 9 days to assess growth.

### RNA isolation

RNA from breast cancer cell lines was isolated using the ReliaPrep™ RNA Cell Miniprep System (Promega, UK) and genomic DNA degraded via DNase I (Promega, UK) treatment according to manufacturer’s instructions. RNA from tissue samples was isolated by homogenization in Trizol followed by centrifugation at 12000xg for 10 min at 4 °C. The supernatant was then phase-separated with chloroform according to manufacturer’s instructions and the upper aqueous phase further purified and treated with DNase I (Qiagen, Manchester, UK) on RNeasy spin columns (Qiagen, UK). Genomic DNA was degraded on the spin columns with DNase I (Qiagen, UK) treatment.

Formalin fixed paraffin embedded samples (8 µm) were heated at 60 °C for 60 min before being placed in xylene for 5 min then 99% ethanol and finally being air dried. De-waxed tissue was scrapped off the slide into micro-centrifuge tubes. These samples were then processed according to the RNeasy^®^ FFPE Kit (Qiagen, UK) manufacturer’s instructions.

### Reverse transcription, conventional PCR and real-time quantitative PCR

Reverse transcription, for conventional PCR, was performed with 1 µg of isolated RNA and using oligo (dT) primers and Superscript II Reverse Transcriptase (Life Technologies, Paisley, UK) following manufacturer’s instructions. PCRs were performed with the cDNA, PCR master mix (Promega, UK) and the following primers; *HER2* exon 8 forward 5'-AACACAGCGGTGTGAGAAGT-3'; *HER2* exon 10 reverse 5'-GTGATCTCTTCCAGAGTCTC-3'; *HER2* exon 12 forward 5'-GGCCAGAGGACGAGTGTG-3', *HER2* exon 14 reverse 5'-CGGTCCAAAACAGGTCACT-3'; β Actin forward 5'-GGACTTCGAGCAAGAGATGG-3'; β Actin reverse 5'-AGCACTGTGTTGGCGTACAG-3'.

For real-time quantitative PCR (RT qPCR), cDNA was produced at 250 nm/µl and diluted as appropriate (Merideian Bioscience Tetro™, Scientific Laboratory Supplies, Hessle, UK). Taqman probes were designed and validated to identify HER2-PI9, HER2-I12 and HER2-WT (Life Technologies by Thermo Fisher Scientific, Cramlington, UK). A mix of 0.5 µl taqman probe, 5 µl Taqman™ gene expression master mix (Life Technologies by Thermo Fisher Scientific, UK), 2.5 µl DNase free H2O and 2 µl cDNA was run on the StepOne Plus real-time PCR system (Life Technologies by Thermo Fisher Scientific, UK), measuring a quantitative CT. Relative quantity was calculated according to the Δ Δ CT method. All RT qPCR used the β-actin housekeeper to normalize data. All data is presented as a mean ± SE. Two repeats were performed and a representative presented in the results section.

### In silico analysis

Expasy Translate tool (available at http://web.expasy.org/translate/) was used to predict amino acid sequences of HER2-PI9 and HER2-I12. Expasy Compute pI/Mw tool (available at http://web.expasy.org/compute_pi/) was used to predict the molecular weight of HER2-PI9 and HER2-I12 proteins.

### Protein isolation and western blot analysis

Proteins were extracted using RIPA buffer (25 mM Tris–HCl pH7.6, 0.1% SDS, 150 mM NaCl, 1% NP-40, 0.1% sodium deoxycholate, 20 µl/ml protease inhibitor cocktail (Sigma-Aldrich, UK), 10 µl/ml of phosphatase inhibitor cocktail (Sigma-Aldrich, UK)). To extract proteins separately, based on subcellular location the subcellular protein fractionation kit for cultured cells was used (Thermo Fisher Scientific, UK), according to manufacturer’s instructions. To separate phosphorylated protein and unphosphorylated protein the phosphoprotein purification kit was used according to manufacturer’s instruction (Qiagen, UK). Following protein quantification, 15 µg or 30 µg of protein was mixed with SDS loading buffer (125 mM Tris–HCl pH6.8, 2% SDS, 10% glycerol, 10% β mercapthoethanol, 0.1% bromophenol blue) and heat denatured at 95 °C for 5 min. Protein lysates were separated using 7.5% polyacrylamide gels for electrophoresis. Subsequently, proteins were transferred onto PDVF membranes (BioRad, Hertfordshire, UK) followed by blocking in 5% milk powder (Sigma-Aldrich, UK) in 1X TBS (Sigma-Aldrich, UK) for 1 h. Afterwards, membranes were incubated overnight at 4 °C with; mouse anti-α-tubulin (T6074, 1:4000, Sigma-Aldrich, UK); rabbit anti-HER2/ERBB2 XP (D8F12, 1:1000, Cell Signalling Technology, Massachusetts, USA); rabbit anti-Akt (C67E7, 1:1000, Cell Signalling Technology, USA); rabbit anti-phospho-Akt (Ser473) (D9E, 1:2000, Cell Signalling Technology, USA); rabbit anti-p44/42 MAPK (9120, 1:1000, Cell Signalling Technology, USA); rabbit anti-phospho-p44/42 MAPK (Thr202-Tyr204) (D13.14.4E, 1:2000, Cell Signalling Technology, USA); rabbit anti-pan-cadherin (GTX132646, 1:5000, Genetex, California, USA); rabbit anti-transferrin (GTX101035, 1:1000, Genetex, USA); rabbit anti-C-jun (GTX134295, 1:500, Genetex, USA); mouse anti-phosphoserine 4A4 (05-1000X, 1:2000, Sigma-Aldrich, UK). Horseradish peroxidase conjugated secondary antibodies (Dako by Aligent, Didcot, UK) were diluted 1:3000 in 1X TBS with 0.01% Tween 20 and incubated for 1 h at room temperature. Protein bands were visualized by chemiluminescence using ECL western blotting substrate (Thermo Fisher Scientific, UK).

### Statistics and data handling

Data from RT qPCR and biological assays were analysed using GraphPad Prism version 8.0.0 for Windows (California, USA, www.graphpad.com). P values less than 0.05 were considered significant.

## Results

### Novel splice variant, HER2-PI9 and HER2-I12, identification

**Fig. 1:**
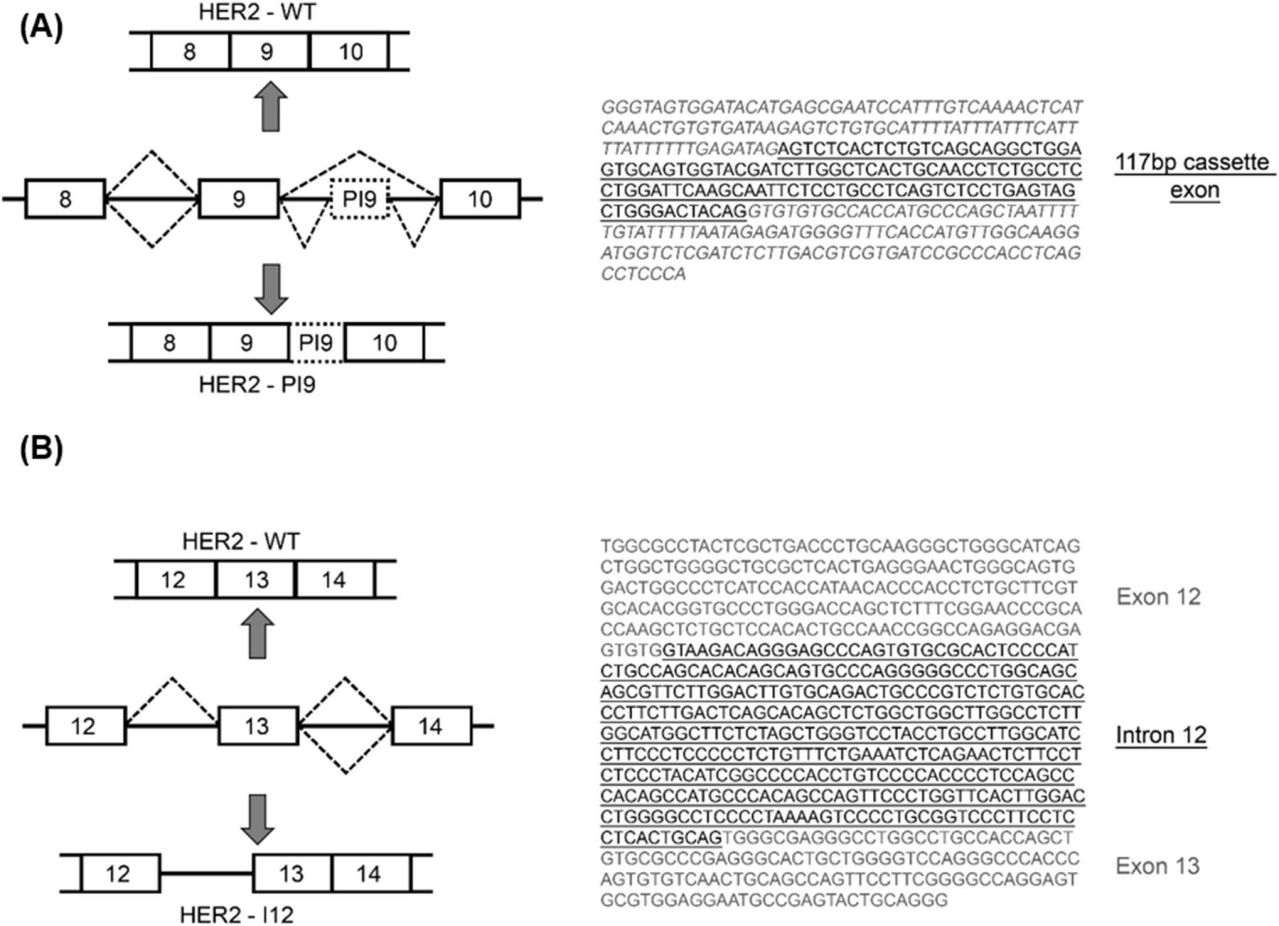
HER2-PI9 and HER2-I12 novel splice variant transcript identification. Conventional PCR was used to screen for alternative splice variants of HER2 in the SKBR3 (HER2 +) cell line. Primers used included; *HER2* exon 8 forward 5'-AACACAGCGGTGTGAGAAGT-3'; *HER2* exon 10 reverse 5'-GTGATCTCTTCCAGAGTCTC-3'; *HER2* exon 12 forward 5'-GGCCAGAGGACGAGTGTG-3', *HER2* exon 14 reverse 5'-CGGTCCAAAACAGGTCACT-3'; β Actin forward 5'-GGACTTCGAGCAAGAGATGG-3'; β Actin reverse 5'-AGCACTGTGTTGGCGTACAG-3'. **A** Alternative splicing produces a variant transcript with partial intron 9 inclusion. The HER2-PI9 variant transcript includes a 117 bp cassette-exon sequence (underlined). **B** Intron 12 (underlined) is retained by alternative splicing to produce the HER2-I12 variant transcript

To identify novel HER2 spliced variants a PCR-based assay was performed on cDNA from SKBR3 cells. Multiple sets of primer pairs were designed to span the exons and introns of the *ERBB2/HER2* gene, allowing exon skipping or intron retention to be detected. The results of this PCR-based screening (Fig. 1) identified two previously unreported alternate splicing events. The first transcript identified, referred to as HER2-partial intron 9 (HER2-PI9), included a 117 bp cassette-exon located within intron 9 of HER2. This was precisely 704 nucleotides downstream from exon 8 and 2016 nucleotides upstream from exon 9 (Fig. 1A). This novel *HER2* transcript was detected with a primer pair that spanned intron 9 (Exon 8 forward primer and Exon 10 reverse primer), as a result these primers could detect both the canonical HER2 isoform (HER2-WT) as well as HER2-PI9 (identity of PCR products were confirmed via sequencing).

The second transcript identified by the PCR screen was produced through the retention of the whole of intron 12 (361 bp) and is referred to as HER2-Intron 12 (HER2-I12) (Fig. 1B). This transcript was detected with a primer pair that spanned the region surrounding intron 12 (Exon 12 forward primer and Exon 14 reverse primer). These primers could detect both the canonical HER2 isoform (HER2-WT) as well as the HER2-I12 transcript, containing a retained intron 12 (identity of PCR products were confirmed by sequencing).

### Relative expression of HER2-WT and novel splice variants in breast cancer cell lines

**Fig. 2:**
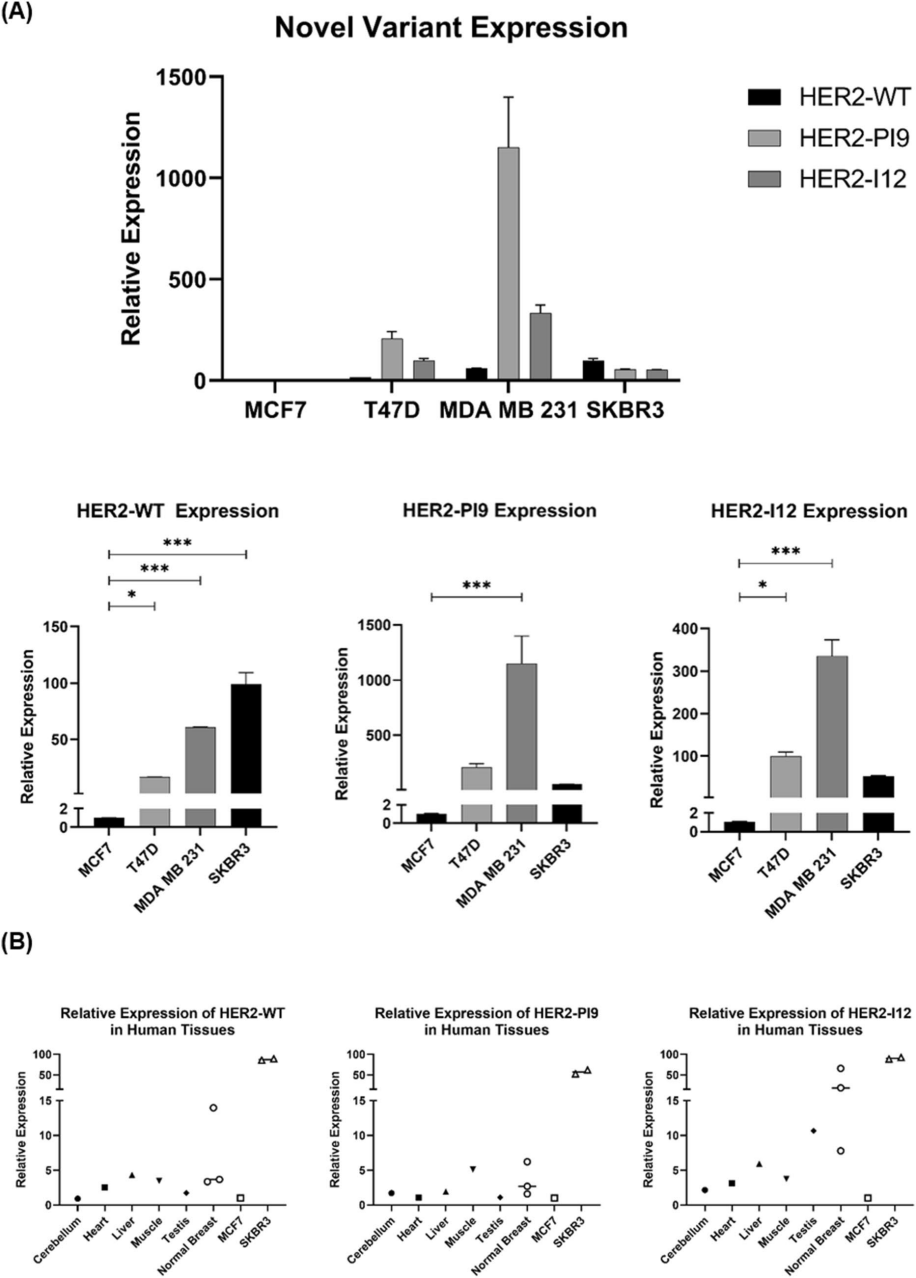
Detection of HER2-PI9 and HER2-I12 splice variants in breast cancer cell lines and tissues. **A** The relative mRNA expression of HER2-WT, HER2-PI9 and HER2-I12 in MCF-7 (HER2-), T47D (HER2 −), MDA-MB-231 (HER2 −) and SKBR3 (HER2 +) breast cancer cell lines. Taqman real-time PRC assays were designed and validated to correspond to each splice variant. HER2-WT was significantly higher in the HER2 + cell line SKBR3, compared to all the HER2- lines including MCF-7 (*p* < 0.0001), T47D (*p* < 0.0001) and MDA-MB-231 (*p* = 0.0002). HER2-PI9 had particularly high relative expression in the cell line MDA-MB-231, compared to the cell lines MCF-7, (*p* < 0.0001), T47D (*p* = 0.0002) and SKBR3 (*p* < 0.0001). HER-I12 expression was significantly higher in T47D compared to MCF-7 (*p* = 0.0247) and MDA-MB-231 compared to MCF-7 (*p* < 0.0001) or SKBR3 (*p* < 0.0001). The line MDA-MB-231 expresses HER-I12 to a greater extent than T47D (*p* < 0.0001). **B** HER2-WT, HER2-PI9 and HER2-I12 mRNA relative expression in human tissues. The HER2 splice variants are all present in cerebellum, heart, liver, muscle, testis and normal breast tissues although no tissue had significantly higher expression than another. Data is normalized to the housekeeper β-actin. Data represents mean ± SE. ***p < 0.001, **p < 0.01, *p < 0.05, one-way ANOVA

RT qPCR was used to identify HER2 splice variant expression in the cell lines MCF-7 (HER2 −/ER +), T47D (HER2 −/ER +), MDA-MB-231 (HER2 −/ER −) and SKBR3 (HER2 + /ER − (Fig. 2A). HER2-WT was significantly higher in the HER2 + cell line, SKBR3 compared to all the HER2 − lines, including MCF-7 (*p* < 0.0001), T47D (*p* < 0.0001) and MDA-MB-231 (*p* = 0.0002). HER2-WT mRNA expression significantly differed between cell lines classified as HER2 −, which is expected as all breast cancer cell lines are heterogeneous in HER2 expression (MCF-7 vs T47D *p* < 0.0159, MCF-7 vs MDA-MB-231 *p* < 0.0001, T47D vs MDA-MB-231 *p* < 0.0001). HER2-PI9 had particularly high relative expression in the cell line MDA-MB-231, compared to the cell lines MCF-7 (*p* < 0.0001), T47D (*p* = 0.0002) and SKBR3 (*p* < 0.0001).

The presence of the HER2-I12 variant was confirmed by RT qPCR within a range of breast cancer cell lines (Fig. 2A). For HER2-I12, relative mRNA expression was significantly higher in T47D compared to MCF-7 (*p* = 0.0247). Expression was also higher in MDA-MB-231 compared to MCF-7 (*p* < 0.0001), SKBR3 (*p* < 0.0001) and T47D (*p* < 0.0001) (Fig. 2A).

Both novel variants, alongside HER2-WT, were also expressed in normal human tissues including cerebellum, heart, liver, muscle, testis, and normal breast tissue (Fig. 2B), expression levels didn’t significantly differ between tissue types.

### Relative expression of HER2 WT and novel variants in breast tumour and normal breast tissue

**Fig. 3:**
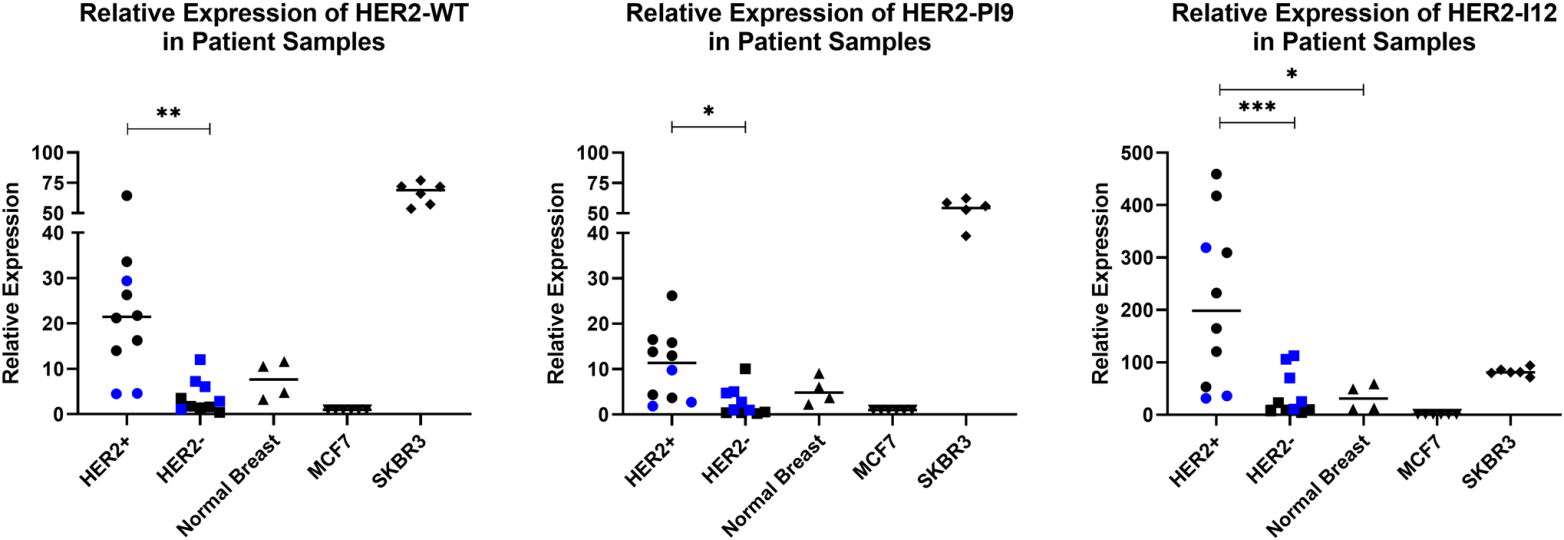
Expression of HER2-PI9 and HER2-I12 splice variants in invasive breast carcinomas. HER2-WT, HER2-PI9 and HER2-I12 variants were identified via RT qPCR in HER2 + (*n* = 10) and HER2 − (*n* = 10) invasive ductal breast carcinomas as well as in normal breast tissue (*n* = 4). Black circles are ER- and blue are ER + . HER2-WT expression was significantly higher in HER2 + tumours compared to HER2 − tumours (*p* = 0.0014). HER2-PI9 was present in HER2 + , HER2 − and normal breast samples. Higher expression was identified in the HER2 + groups compared to the HER2 − group (*p* = 0.0259). HER2-I12 was also present in all groups and was significantly higher in the HER2 + groups compared to HER2 − (*p* = 0.0009) and normal breast (*p* = 0126). ER status did not significantly correlate with variant expression. Data is normalized to the housekeeper β-actin. Data represents mean ± SE. ***p < 0.001, **p < 0.01, *p < 0.05, one-way ANOVA

Expression of these novel variants was also assessed in grade 3 invasive ductal carcinomas. Tissues had previously been assessed for their HER2 status in accordance with clinical guidelines. RT qPCR assays to detect the novel splice variants were performed in normal breast samples (*n* = 4), and HER2 + (*n* = 10) and HER2 − (*n* = 10) samples (Fig. 3). HER2-WT expression was significantly higher in HER2 + tumours compared to HER2- tumours (*p* = 0.0014). HER2-PI9 was present in HER2 + , HER2 − and normal breast samples. Higher expression was identified in the HER2 + groups compared to the HER2 − group, (*p* = 0.0259). HER2-I12 was also present in all groups and was significantly higher in the HER2 + groups compared to HER2 − (*p* = 0.0009) and normal breast (*p* = 0.0126). Expression levels did not significantly change between ER + and ER − patients. Variation between the splice variant expressions is high between patients, even in the same IHC grouping. As a preliminary data set the sample size is too small to draw definitive conclusions, aside from the expression of the novel variants occurring in patients, and will be expanded.

### Investigating HER2-PI9 and HER2-I12 via in silico analysis and recombinant HER2-PI9 and HER2-I12 expressed in MCF-7 cell line

**Fig. 4:**
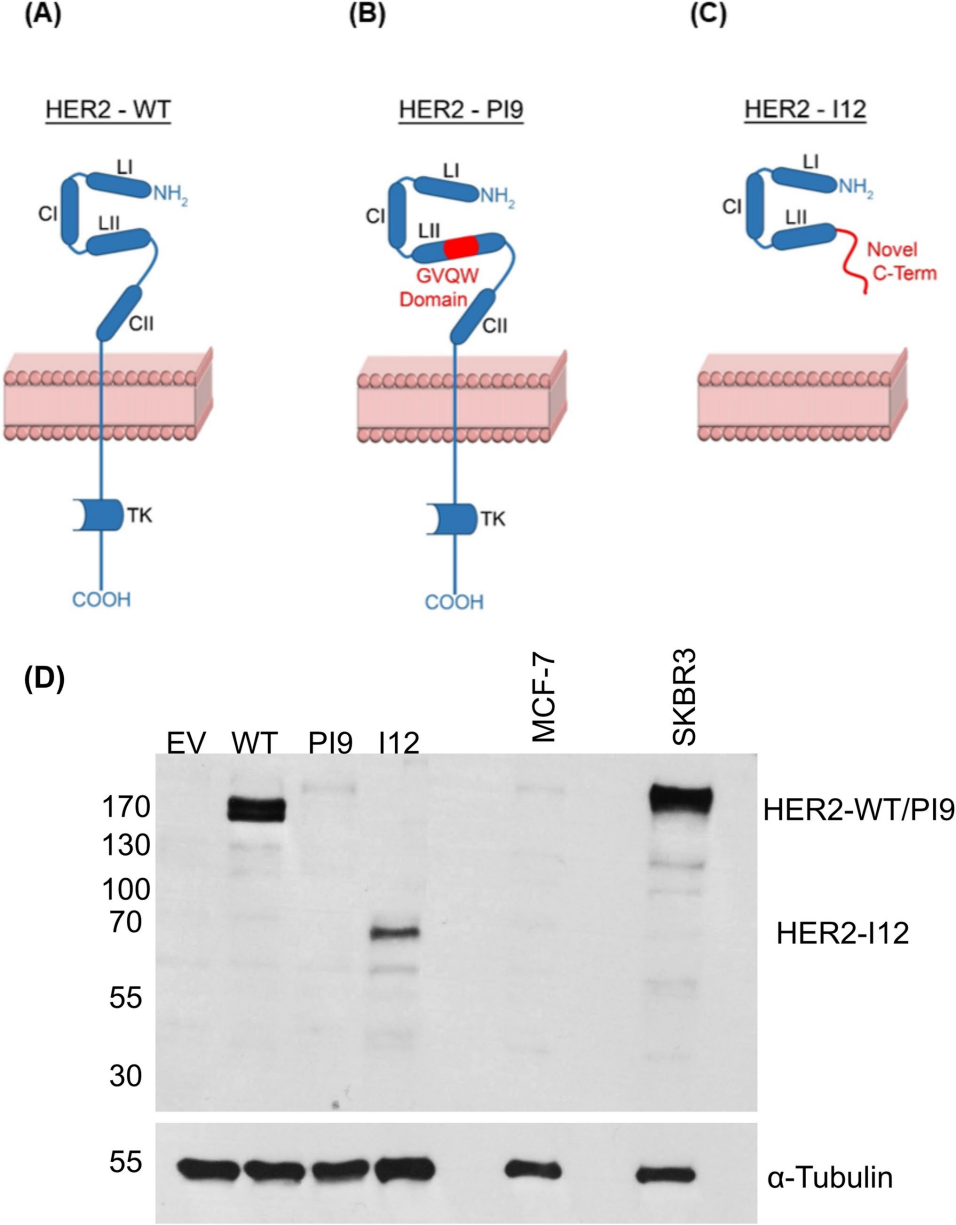
HER2-PI9 and HER2-I12 predicted protein structures and in-vitro model production. In silico predictions of protein structure for the novel splice variants based off their RNA sequence using the Expasy Translate Tool software. **A** HER2-WT protein structure showing the LI, LII, CI and CII extracellular domains and the intracellular tyrosine kinase (TK) domain. *NH*_2_ amino-terminus. *COOH* carboxyl-terminus. **B** HER2-PI9 predicted protein structure including the GVQW domain (highlighted red). **C** HER2-I12 predicted protein structure including the novel C-terminal region (highlighted red). **D** Detection by western blotting of HER2-WT, HER2-PI9 and HER2-I12 proteins in MCF-7 cells transformed with expression vectors. HER2-WT and HER2-PI9 are present above the 170 kDa protein marker, correlating to the 185 kDa and 190 kDa predicted proteins respectively. HER2-I12 has a band just below the 70 kDa marker, correlating to the predicted 64 kDa protein. The empty vector control has protein expression relative to the MCF-7 control. The cell line, SKBR3, was included as a positive control as it expresses high levels of HER2-WT. 30 μg total protein was loaded in each lane. Alpha-tubulin (50 kDa) was blotted for as a loading control

To predict the amino acid sequences of HER2-PI9 and HER2-I12 and to compare these with HER2-WT, in silico analysis was performed using the Expasy Translate Tool software (Fig. 4A). HER2-PI9 protein (predicted molecular weight 190 kDa) might preserve the full amino acid sequence of HER2-WT, with an additional novel 39 amino acids located within the LII domain. These additional residues comprise a GVQW putative binding domain (Fig. 4B). The exact role of this domain is not completely understood, however this motif is found in proteins which regulate apoptosis, such as caspases (Howald et al. 2012), as well as DNA repair, including BRIP1 (BRCA1 interacting protein C-terminal helicase 1) (Ouhtit 2016). HER2-I12 was predicted to generate a truncated HER2 protein (predicted molecular weight 64 kDa) because of a premature termination codon located 212 nucleotides downstream of the start of intron 12 of *ERBB2*. HER2-I12 therefore should contain 504 N-terminal amino acids which are homologous to HER2-WT, with a novel 73 C-terminal residues located downstream of the LII domain (Fig. 4C).

To investigate HER2-PI9 and HER2-I12 potential protein expression, three plasmid expression vectors were generated. A HER2 vector (HER2-WT) contained the HER2 coding DNA sequence (CDS); a HER2-I12 vector contained the HER2 CDS plus intron 12 of *HER2*; and finally, a HER2-PI9 vector contained the HER2 CDS along with the 117 bp cassette-exon from intron 9 of *HER2*. MCF-7 cells were transfected with the HER2-WT, HER2-PI9 and HER2-I12 expression vectors. Stable lines were produced and protein expression confirmed by Western blotting (Fig. 4D). MCF-7 cells were used in this experiment to exclude detection of endogenous HER2 proteins, as they are a HER2 negative breast cancer cell line and so express low amounts of HER2-WT. As expected, the HER2 N-terminal antibody was unable to detect HER2 protein expression in the untreated MCF-7 control lane but did detect protein bands in the cells transfected with the three expression vectors. Cells transfected with the HER2-PI9 expression vector produced a protein band at around 190 kDa, and those transfected with HER2-I12 produced a protein band at around 64 kDa, which matched the predicted molecular sizes obtained from in silico analysis.

### Cellular location of HER2 proteins

**Fig. 5:**
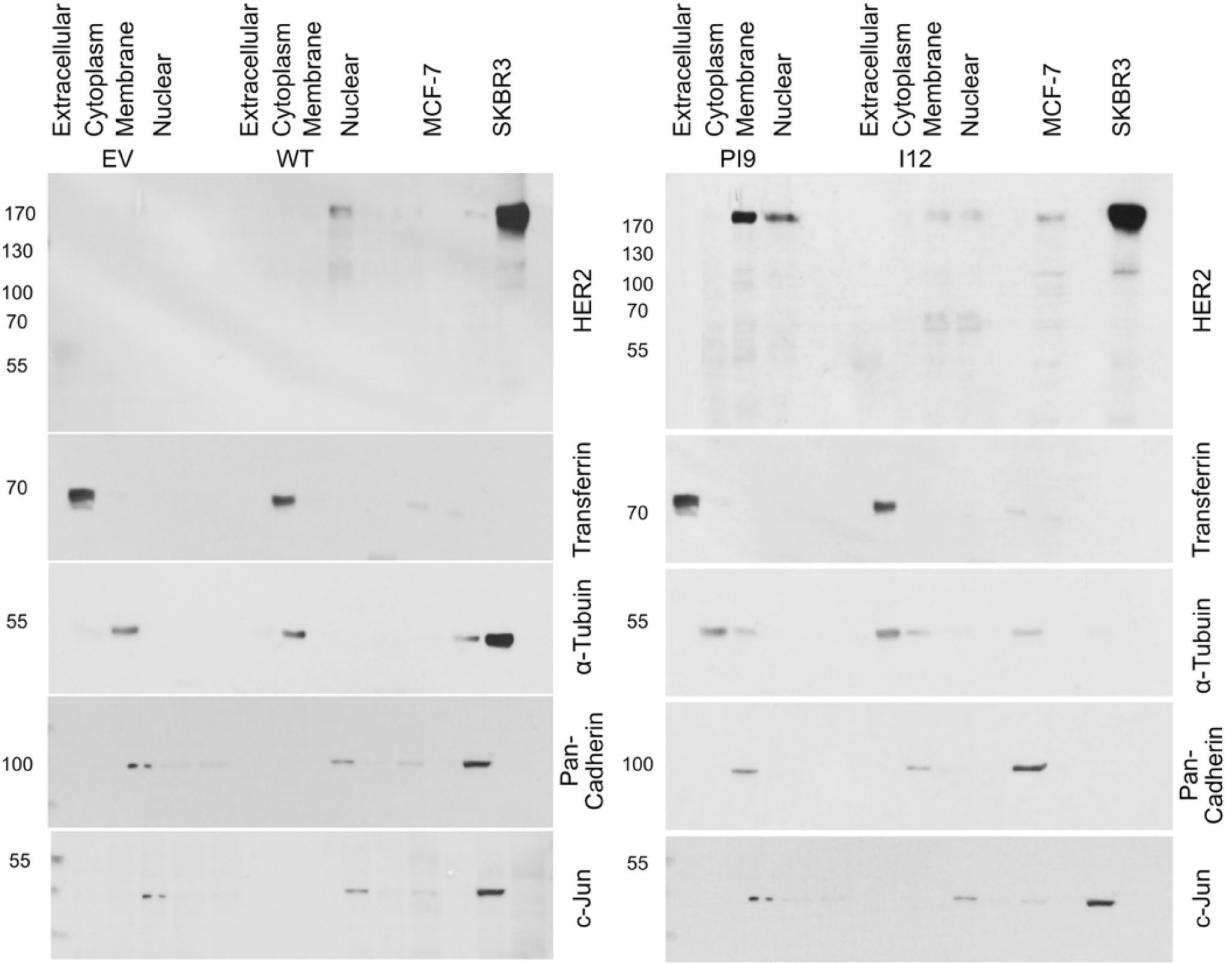
Expression of HER2-PI9 and HER2-I12 variants in cellular fractions. Western blot analysis of protein extracted from the cell growth media (extracellular), cytoplasm, membrane and nucleus. **A** Protein levels of the empty vector (EV) control and HER2-WT. For the EV control no HER2 protein was identified. HER2-WT (185kDA) was only expressed in the membranous fraction. **B** Protein levels of HER2-PI9 and HER2-I12. HER2-PI9 (190 kDa) is expressed in the membranous and nuclear fractions. HER2-I12 (64 kDa) is expressed in the membranous and nuclear fractions. 30 μg total protein was loaded in each lane. Multiple loading controls were included with each specifically expressed in a single fraction to ensure protein was only extracted from the intended cellular compartment. Loading controls included transferrin (77 kDa) (extracellular marker), α-tubulin (50 kDa) (cytoplasmic marker), pan-cadherin (100 kDa) (membrane marker) and c-jun (36 kDa) (nuclear marker) as loading controls. This experiment was run thrice and a representative image shown

To confirm these variants are fully mature transcripts transfected MCF-7 cells were fractionated to isolate the cytoplasmic, membranous, and nuclear fractions. Protein was extracted from the culture media to obtain extracellular, secreted, proteins. Western blot analysis using an anti-HER2 antibody identified the fractions in which specific splice variants were present (Fig. 5). No HER2 protein was present in the EV control cell line. HER2-WT was identified as present at the membrane only. HER2-PI9 was present at the membrane and nuclei, to a lesser extent. HER2-I12 exhibited expression at the membrane and the nucleus. HER-I12 samples show HER2-WT expression at 185 kDa comparable to the basal MCF-7 expression, alongside the 64 kDa variant. Western blot analysis only identified transferrin protein in the extracellular lanes, α-tubulin in the cytoplasmic fraction, pan-cadherin in the membranous fraction only and c-jun in the nuclear fraction indicating fractionation was successful (Fig. 5).

**Fig. 6:**
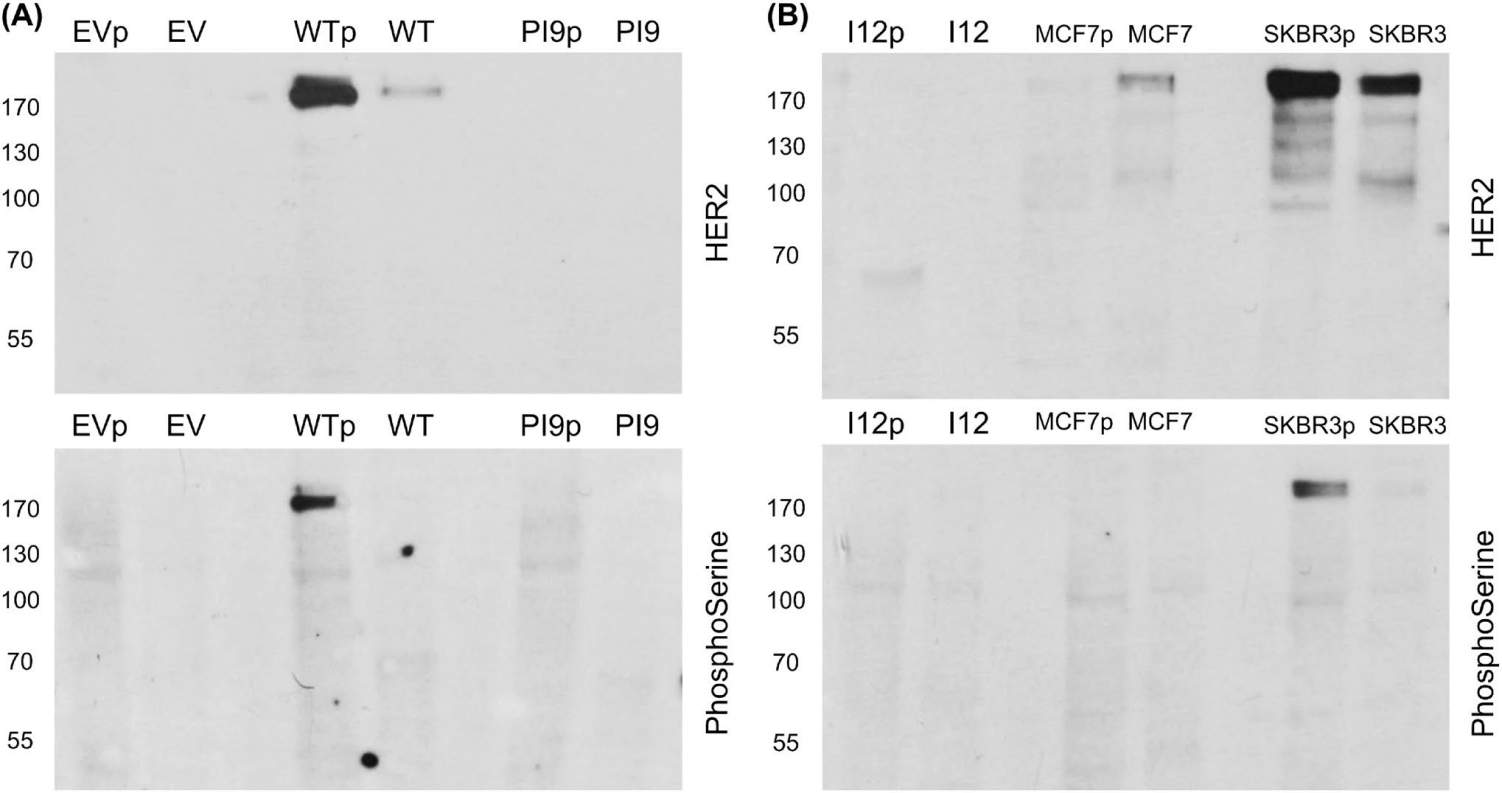
Phosphorylation status of HER2-WT, HER2-PI9 and HER2-I12. Western blot analysis of segregated phosphorylated and non-phosphorylated protein extracted from cells expressing either an empty vector control (EV), HER2-WT, HER2-PI9 or HER2-I12. Protein from the MCF-7 (HER2 −) and SKBR3 (HER2 +) cell lines were included as to show weak and strong phosphorylation of HER2 proteins. **A** An anti-HER2 antibodies identifies strong phosphorylation of HER2 in the HER2-WT cell line which is lacking in the EV control and PI9 cell line. **B** HER2-I12 and SKBR3 cell lines both show strong HER2 phosphorylation. 15 μg total protein was loaded in each lane. Phosphoserine was used as a loading control to identify proteins in the phosphorylated fraction. This experiment was performed twice and a representative image shown

### HER2-PI9 and HER2-I12 expression and activation status influence PI3K/Akt and RAS/MAPK signalling pathway in MCF-7 cells

The phosphorylation status of the HER2 splice variants was considered by extracting phosphorylated and non-phosphorylated protein in cell lysate separately. HER-WT and HER2-I12 lines showed relatively large amounts of phosphorylated protein compared to non-phosphorylated, implying an activated status (Fig. 6). HER2-PI9 did not show phosphorylation. The HER2 − cell line showed weak staining for endogenous HER2 expression, and no phosphorylation. The SKBR3 cell line expressed large amounts of HER2 in both phosphorylated and non-phosphorylated states.

**Fig. 7:**
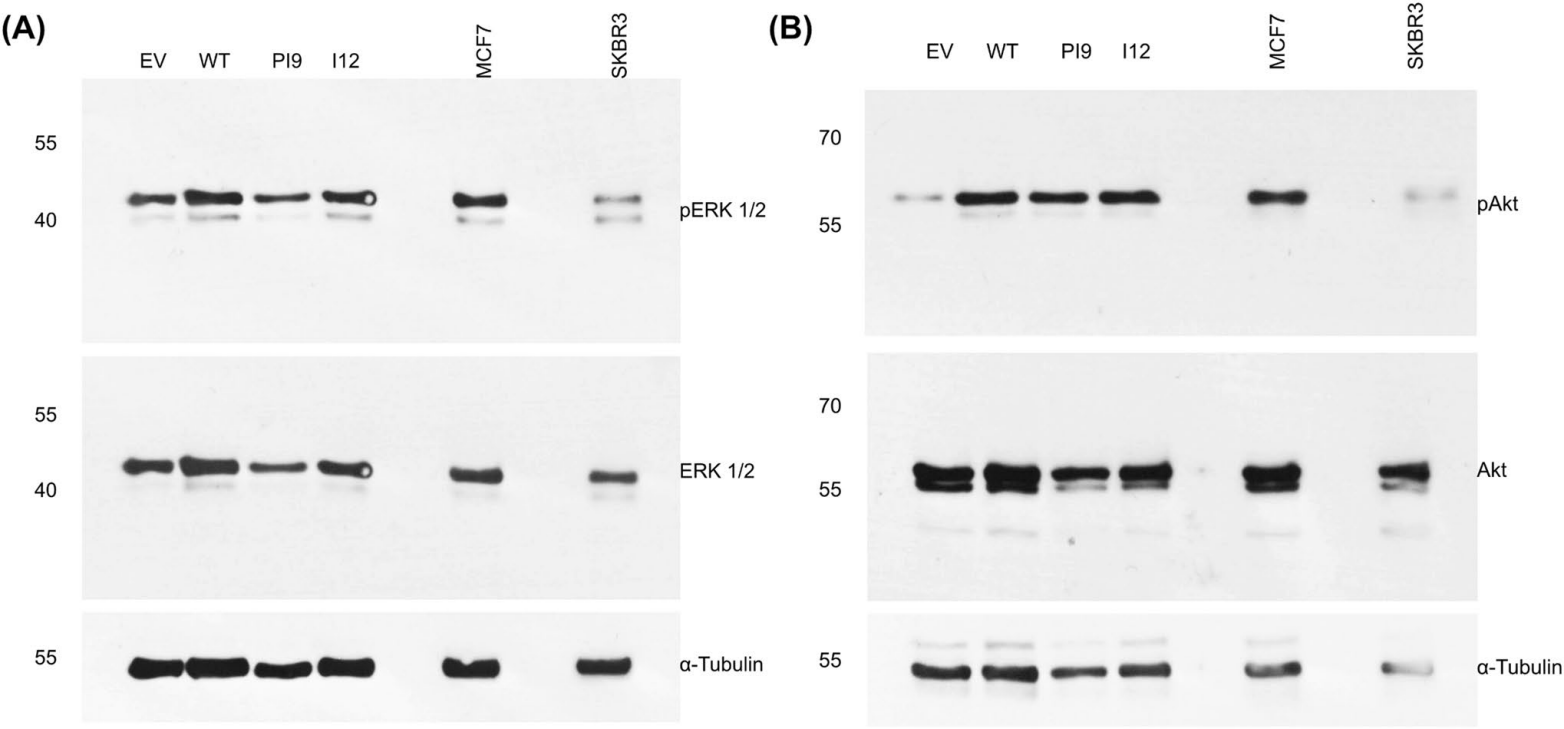
Effects of HER2-PI9 and HER2-I12 expression on ERK1/2 and AKT phosphorylation. Western blot analysis of the activation of the HER2 associated signalling pathways, PI3K/Akt and RAS/MAPK following transfection of MCF-7 cells with the empty vector control (EV), HER2-WT, HER2-PI9 and HER2-I12 expression vectors. **A** Phosphorylation of Akt (60 kDa) indicates activation of the PI3K/Akt pathway. HER2-WT or HER2-I12 expression activates Akt, shown by increased phosphorylation. **B** Phosphorylation of ERK 1/2 indicates activation was caused by HER2-WT or HER2-I12 expression. 30 μg total protein was loaded in each lane. Alpha-tubulin was used as a loading control (50 kDa). This experiment was run thrice and a representative image shown

Western blot analysis assessed expression and phosphorylation of ERK1/2 (RAS/MAPK pathway) and Akt (PI3K/Akt pathway) to identify activation (Fig. 7). Results indicated that the ERK1/2 levels, as well as Akt, were consistent amongst all the samples. Transfection of the cells with HER2-WT resulted in increased phosphorylation of both ERK1/2 (pERK1/2) and Akt (pAKT), indicating activation. In addition, cells transfected with HER2-I12 and, to a lesser extent, HER-PI9 also showed increased phosphorylation of ERK1/2 (pERK1/2) and AKT (pAKT).

**Fig. 8:**
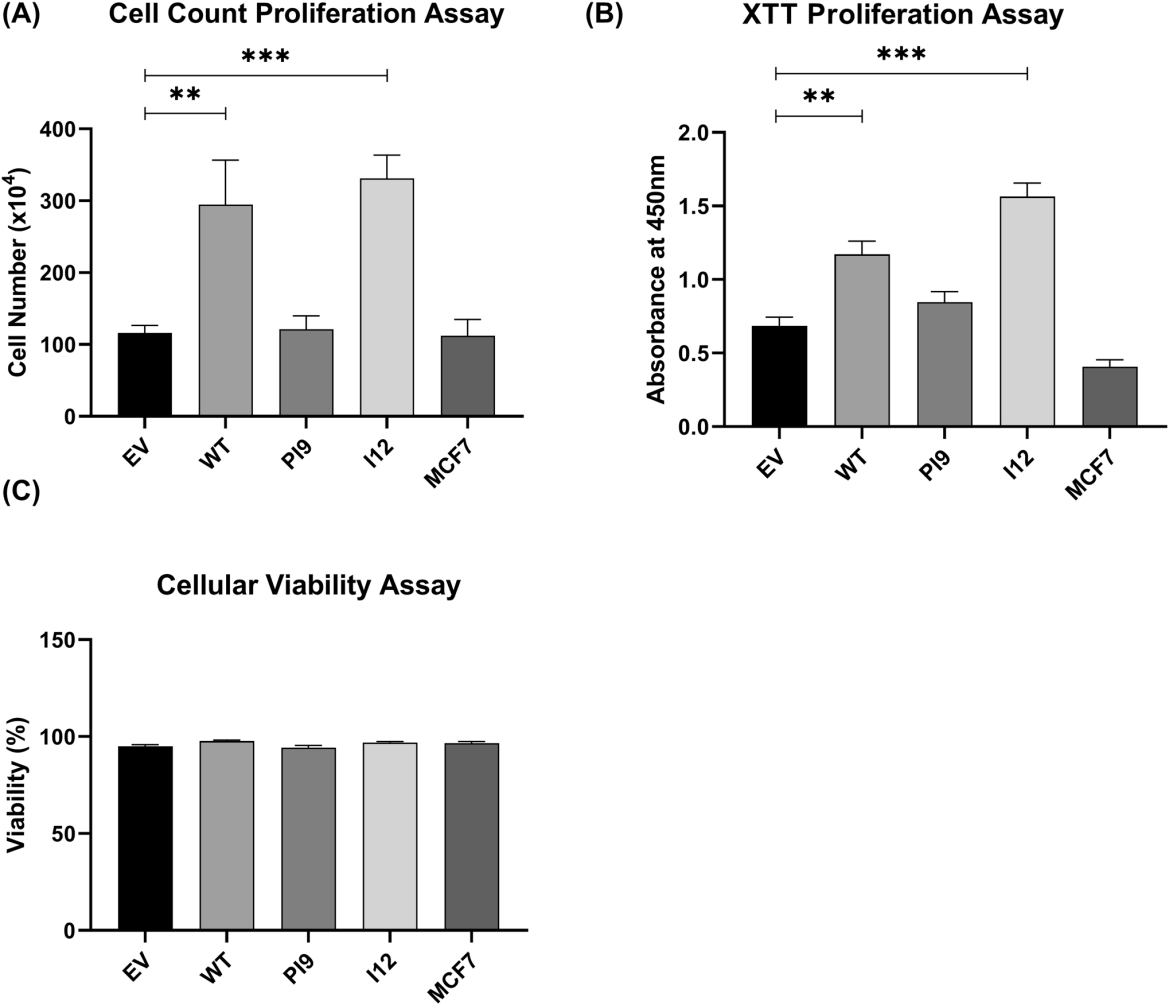
Effect of novel splice variant expression on proliferation and viability. Cell lines were produced from the HER2- cell line MCF-7, to express HER2-WT, HER2-PI9 or HER2-I12. **A** An XTT assay was used to analyse the effect of splice variant expression on cellular proliferation. **B** and** C** A trypan blue assay was also used to assess the effect of splice variant expression on proliferation and viability. The expression of HER2-WT and HER2-I12 enhanced proliferation significantly. No effect on viability was identified. Data represents mean ± SE. ***p < 0.001, **p < 0.01, *p < 0.05, two-way ANOVA (*n* = 3)

### HER2-PI9 and HER2-I12 expression influence on cellular phenotype

**Fig. 9:**
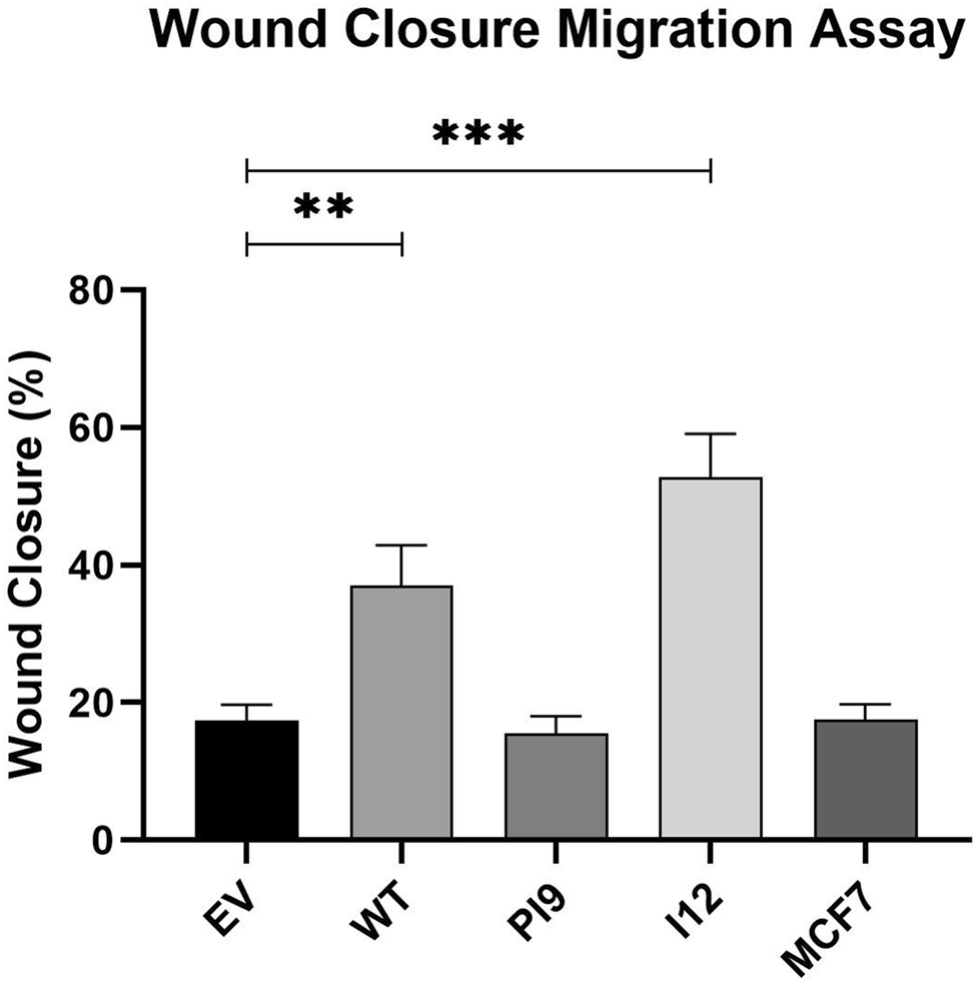
Effect of novel splice variant expression on cellular migration. Cell lines were produced from the HER2 − cell line MCF-7, to express HER2-WT, HER2-PI9 or HER2-I12. A scratch assay was used to assess migration of the cells. Confluent cells were disrupted with a 200 μl pipette tip to produce a scratch devoid of adherent cells. At 48 h the percentage wound closure was calculated. HER2-WT and HER2-I12 expression enhanced migratory ability of the cells. Data represents mean ± SE. ***p < 0.001, **p < 0.01, *p < 0.05, two-way ANOVA (*n* = 3)

As the novel variants enhanced signalling through the RAS/MAPK and PI3K/Akt pathways, stable cell line models were utilised to investigate impact upon cellular phenotypes. Proliferation of cells were enhanced by HER2-WT and HER2-I12 splice variant expression (Fig. 8A, B). HER-PI9 did not enhance proliferation, despite correlating with increased phosphorylation of Akt and ERK 1/2. Cell viability was not affected by expression of either of the splice variants (Fig. 9C).

**Fig. 10:**
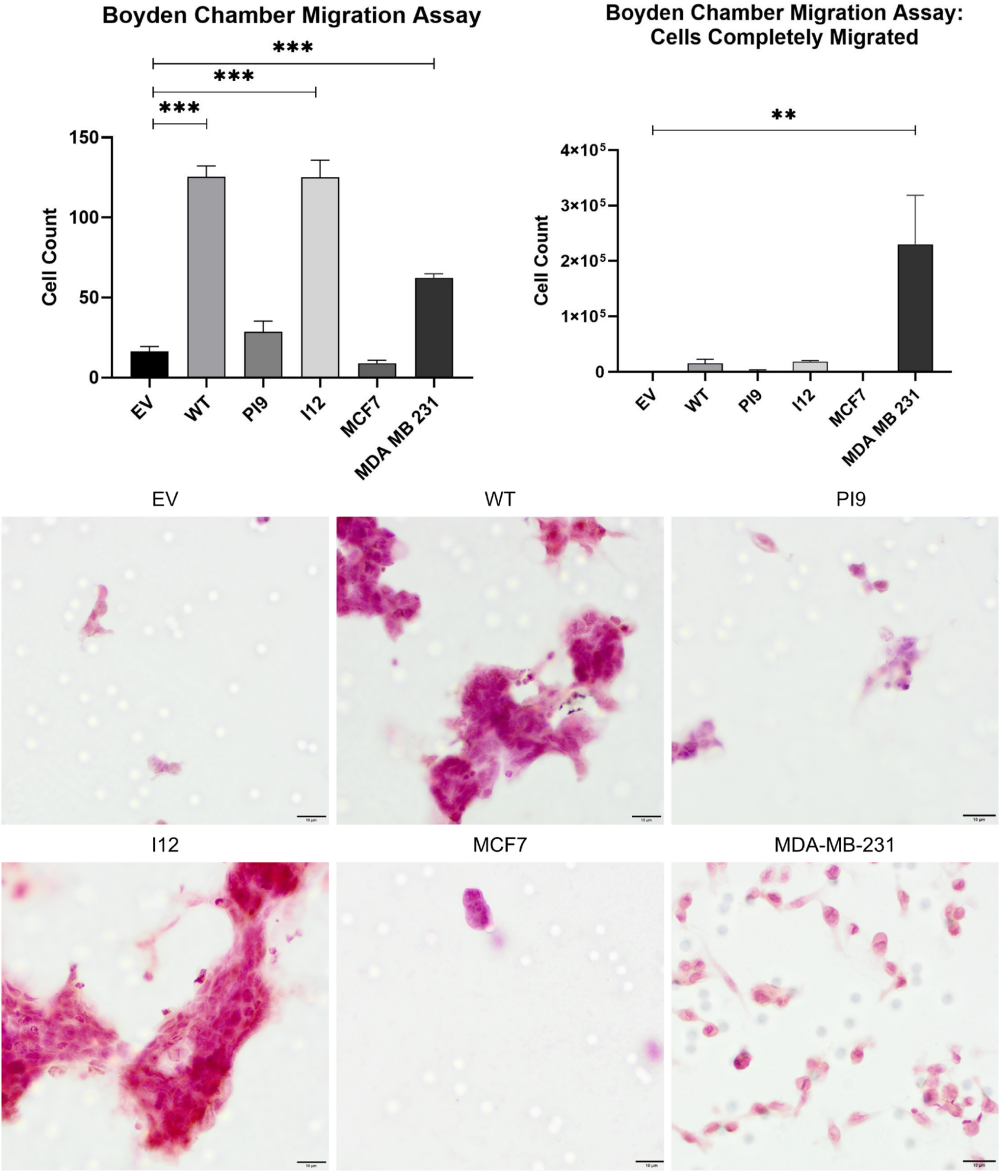
Effect of novel splice variant expression on migration. Cell lines were produced from the HER2- cell line MCF-7, to express HER2-WT, HER2-PI9 or HER2-I12. A boyden chamber assay was completed to assess migratory ability. The HER2 − cell line MDA-MB-231 was included as a comparison as an invasive cell line. Serum starved cells were seeded in the upper chamber and 20% foetal bovine serum-containing media was used as a chemoattractant in the lower chamber. At 24 h the cells on the transwell were fixed and stained with Mayer’s Hematoxylin Solution to assist counting. Cells that had migrated completely through the chamber were also counted. Scale bars indicate 200 μM. HER2-WT and HER2-I12 expression enhanced migratory ability of the cells. The invasive line MDA-MB-231 were also able to migrate through the boyden chamber to a greater extent than the MCF-7 cells. Data represents mean ± SE. ***p < 0.001, **p < 0.01, *p < 0.05, two-way ANOVA (*n* = 3)

**Fig. 11:**
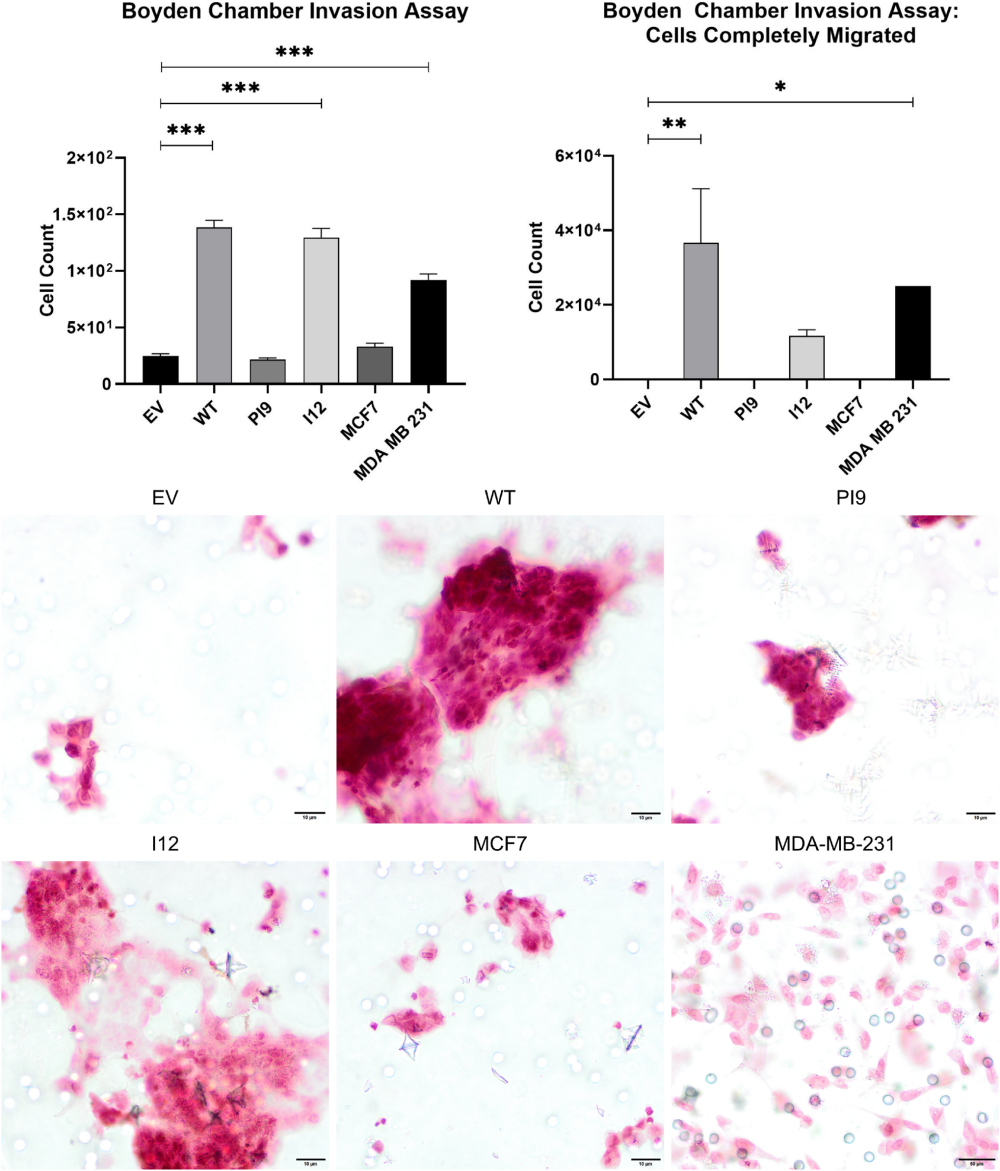
Effect of novel splice variant expression on invasion. Cell lines were produced from the HER2 − cell line MCF-7, to express HER2-WT, HER2-PI9 or HER2-I12. A boyden chamber assay was completed to assess invasive ability. The HER2- cell line MDA-MB-231 was included as a comparison as an invasive cell line. Serum starved cells were seeded in the upper chamber atop of an extracellular matrix and 20% foetal bovine serum-containing media was used as a chemoattractant in the lower chamber. At 24 h the cells on the transwell were fixed and stained with Mayer’s Hematoxylin Solution to assist counting. Cells that had migrated completely through the chamber were also counted. Scale bars indicate 200 μM. HER2-WT and HER2-I12 expression enhanced invasive ability of the cells. The invasive line MDA-MB-231 were also able to migrate through the boyden chamber to a greater extent than the MCF-7 cells. Data represents mean ± SE. ***p < 0.001, **p < 0.01, *p < 0.05, two-way ANOVA (*n* = 3)

**Fig. 12:**
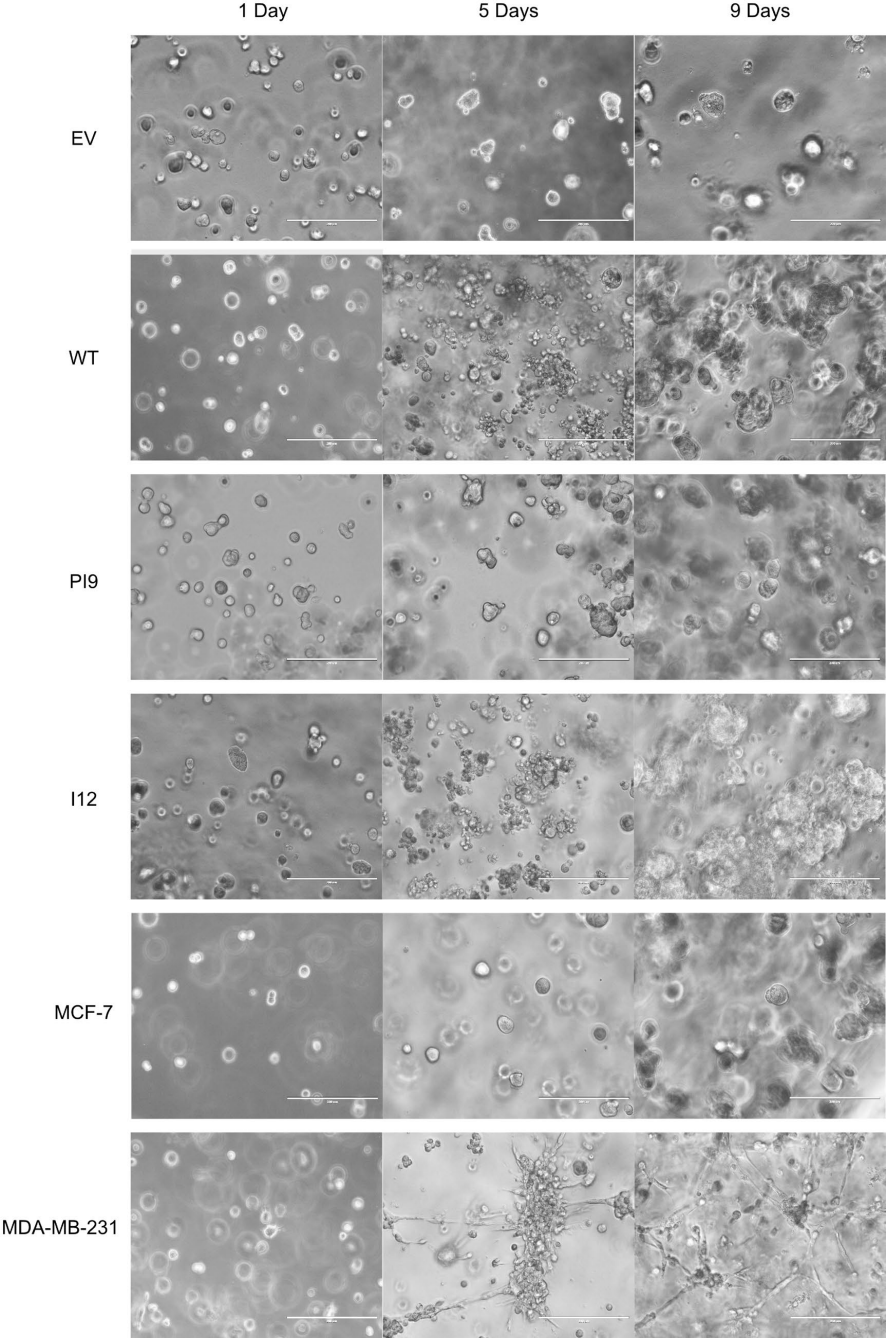
3D culturing of HER-WT, HER2-PI9 and HER2-I12 expressing cell lines. Cell lines were produced from the HER2 − cell line MCF-7, to express HER2-WT, HER2-PI9 or HER2-I12 and grown in 3D cultures. An extracellular matrix layer coated a plate to halt adherence. Cells were embedded within a 10% extracellular matrix layer with a feeding layer of complete media to ensure nutrient delivery into the system. A final cell density of 3 × 10^5^ was embedded in each well. Pictures shown were taken 24 h after seeding and nine days after seeding. Scale bars indicate 200 μM

A scratch assay indicated that HER2-WT and HER2-I12 enhance migration (Fig. 9). This observation was strengthened using a Boyden chamber assay as HER2-WT and HER2-I12 models migrated to a greater extent compared to the EV control and HER2-PI9 (Fig. 10). The HER2-WT and HER2-I12 splice variant cell models also were able to invade through an extracellular matrix, unlike the HER-PI9 model (Fig. 11). When grown in a 3D extracellular matrix, HER2-WT and HER2-I12 appeared to grow larger cultures compared to the empty vector control, HER2-PI9 and MCF-7 cells (Fig. 12).

## Discussion

In this study, two novel splice variants of the oncogene ERBB2/HER2 have been identified. HER2-PI9 contains an 117 bp cassette-exon located within intron 9 of HER2*.* The predicted sequence only differs to HER2-WT with the addition of a novel 39 amino acid sequence located within the LII domain that comprise a GVQW putative binding domain. HER-I12 includes the entire intron 12 and is predicted to include a premature termination codon and a novel C-terminus after the LII extracellular domain. These splice variants produce protein products of which sizes match predicted sequences.

The Ensembl database (Ensembl release 103 (Hunt et al. 2018)) was searched to identify intron 12 and partial intron 9 retention in annotated ERBB2 transcripts. In both cases, retention has been previously annotated, but the transcripts identified do not match the novel variants detailed above. PI9 retention was found in a protein-encoding transcript (ENST00000578502.1). Intron 12 retention was identified in two transcripts labelled as non-protein coding (ENST00000582788.5 and ENST00000583038.5). Retention of these introns can occur in ERBB2 transcripts that are then differently processed to produce alternative transcripts.

HER2-WT exerts its influence over cellular proliferation and other phenotypes via downstream signalling pathways, predominantly RAS/MAPK and PI3K/Akt (Aksamitiene et al. 2012; Yang et al. 2016). Therefore, assessment of the novel variants’ activation status and ability to trigger cell signalling was essential in establishing functional differences to HER2-WT. Cellular phenotypes associated with HER2 oncogenic abilities were also assessed to characterise the novel variants functionality.

The findings presented show that HER2-PI9 was not highly phosphorylated and was less able to activate both the RAS/MAPK and PI3K/Akt pathways than HER2-WT. The predicted protein sequence is relatively conserved to the HER2-WT receptor and HER2-PI9 was mostly reserved to the membrane, with some expression in the nucleus. The slight change in sequence may somewhat block phosphorylation or activation of the receptor.

Although HER2-I12 was predicted to lack the tyrosine kinase domain, the 64 kDa protein product is phosphorylated and can activate both RAS/MAPK and the PI3K/Akt signalling pathways *in-vitro*. This finding is made less surprising by the relatively large amounts of protein present at the membrane, perhaps implying it can bind to the cellular membrane or dimerize with other family members with the novel C-terminus. Data suggests that HER2-I12 may behave differently to the two previously identified truncated HER2 splice variants, Herstatin and P100; both block downstream signalling likely by binding to HER2-WT receptors at the cell membrane to halt dimerization (Jackson et al. 2013; Silipo et al. 2017).

Downstream effects of the splice variants differed; HER2-I12 expression enhanced cellular proliferation, migration and invasion. HER2-I12 was also able to develop larger colonies in 3D cultures. The behaviour of the HER2-I12 overexpressing model was comparable to that of HER2-WT. This provides further evidence that this is a functional protein that does not follow the pattern of previously identified HER2 truncated isoforms. HER2-PI9 however, did not exhibit the same effect upon cellular properties indicating a less or different functional role for this variant. The effect of these splice variants on further hallmarks of cancer remains to be established.

Both HER2-PI9 and HER2-I12 are also present in the nucleus. Tyrosine kinase receptors have been identified in the nucleus, often after being transported from the membrane surface. When present there they are associated with alterations to transcriptional activities, cell proliferation and DNA repair (Wang et al. 2010). Moreover, nuclear HER2 has been associated with nuclear translocation and the formation of complexes with COX-2 promoters, Cdc2 and STAT3, impacting upon transcription (Wang et al. 2004; Béguelin et al. 2010; Tan et al. 2002). There is evidence that in HER2 + /HR − tumours nuclear HER2 is a predictor of worse overall survival (Schillaci et al. 2012). HER2-PI9, having the GVQW domain, may be involved in apoptosis within the nucleus (Howald et al. 2012). Whilst both novel variants appear to retain the same functioning as HER2-WT, in relation to the activation of signalling, their appearance in the nucleus may suggest additional functional roles.

Both HER2 novel splice variants are present in a range of human tissues, including in normal and cancerous breast. This screen is in its preliminary stages and an increase in sample size and tumour types/grades will need to be undertaken to further explore their expression in tumours and any correlation to tumour behaviour and response to treatment.

The antibody used during IHC assessment of patient’s biopsies, in the UK, is specific to the C-terminal region of the HER2 receptor (Tsai et al. 2019). The HER2 IHC score then given to a patient, which will be used to help decide treatment strategies, will not acknowledge the collective levels of all HER2 isoforms (Cardoso et al. 2018). HER2-I12 is a truncated variant and so will be excluded from a patient’s current IHC HER2 status. As it can activate the same signalling pathways and enhance the same oncogenic properties as HER2-WT, it may contribute to a false negative HER2 status being ascribed to a patient (Ruiz-Saenz et al. 2018). HER2-PI9 likely is identified by the C-terminal antibody used commercially and so will be acknowledged in the IHC HER2 score.

Despite the great improvements to prognosis for patients after the introduction of HER2 targeting agents, resistance to these HER2 targeting treatments remains a problem (Choong et al. 2020). Trastuzumab targets the CII domain of the extracellular region of the HER2 receptor and lapatinib the tyrosine kinase domain located on the intracellular region (Marti et al. 2020). As HER2-I12 has an altered C-terminus, and lacks the intracellular region completely, it is likely these drugs will not be able to target this variant and as such these treatment options may not reduce signalling as completely as desired, if this variant is present. Pertuzumab targets the HER2 receptor at the CI region, a site further from the C-terminus and as such likely still can target HER2-I12 (Nami et al. 2019). HER2-PI9 has mostly retained the HER-WT protein sequence and so can likely be targeted by these treatments. However, the addition of the GVQW sequence and location in the nucleus has unknown consequences that may influence drug resistance. The deeper our understanding of the extent of HER2 splice variant expression, and how these alter cancer cell behaviour, the more accurately a patient can be diagnosed and treatment response be predicted.

## Conclusion

Two novel splice variants of HER2, HER-PI9 and HER-I12, have been identified arising from partial and full intron inclusion. These transcripts produce proteins that are located at the cell membrane as well as within the nucleus. HER2-I12 can activate key oncogenic signalling pathways, namely RAS/MAPK and PI3K/Akt. HER2-I12 also enhances oncogenic properties associated with HER2 overexpression. Determining the precise functionality of these variants during oncogenic transformation and how they respond to HER2 targeting treatments will provide more detail as to how HER2 influences tumour phenotype and drug resistance. More detailed testing during IHC biopsy assessment may allow for a more comprehensive understanding of an individual’s tumour and likely benefit from different targeted therapies.

## Data Availability

All data generated is available.

## Abbreviations

Akt: Akt/protein kinase B
BRIP1: BRCA1 interacting protein C-terminal helicase 1
CDS: Coding DNA sequence
DMEM: Dulbecco’s modified eagle medium
EGFR: Epidermal growth factor receptor
ER: Oestrogen receptor
ERK 1/2: Extracellular signal-related kinase 1/2
EV: Empty vector
FBS: Foetal bovine serum
G418: Geneticin
HER2-: HER2 negative
HER2: Human epidermal growth factor receptor 2
HER2 +: HER2 positive
HER2-I12: HER2 intron 12
HER2-PI9: HER2-partial intron 9
HER2-WT: HER2 wild-type
HER3: Human epidermal growth factor receptor 3
HER4: Human epidermal growth factor receptor 4
IHC: Immunohistochemistry
PBS: Dulbecco’s phosphate-buffered saline
PI3K/Akt: Phosphatidylinositol 3-kinase/protein kinase B
PR: Progesterone receptor
RAS/MAPK: Rat sarcoma/mitogen-activated protein kinase
RT qPCR: Real-time quantitative PCR
XTT: Cell proliferation kit II (XTT, Roche Diagnostics, Mannheim, Germany)
